# Remote vital signs data monitoring during home blood transfusion: A pilot study

**DOI:** 10.1002/hsr2.380

**Published:** 2021-09-14

**Authors:** Akinori Nishikawa, Yoshihiro Fujimori, Noriko Sakano, Toshiki Mushino, Shinobu Tamura, Shingo Kasahara, Hiroshi Akasaka, Takashi Sonoki

**Affiliations:** ^1^ Division of Blood Transfusion Wakayama Medical University Hospital Wakayama Japan; ^2^ Department of Hematology/Oncology Wakayama Medical University Hospital Wakayama Japan; ^3^ Division of Medical Information Wakayama Medical University Hospital Wakayama Japan; ^4^ Akasaka Clinic Kobe Japan; ^5^ Division of Hematology, Department of Internal Medicine Department of Transfusion Medicine and Cellular Therapy Hyogo College of Medicine Nishinomiya Japan; ^6^ Department of Cardiovascular Surgery Okayama University Graduate School of Medicine Okayama Japan

**Keywords:** home blood transfusion, remote monitor, transfusion‐related complication, vital sign data

## Abstract

**Background and aims:**

Our study aimed to establish safer methods to manage home blood transfusion by using a remote vital signs data monitoring system. Home care is administered for patients with various medical disorders; however, home blood transfusion remains challenging owing to the risk of transfusion‐related complications.

**Methods:**

We set up a remote vital signs data monitoring system to improve the safety of home blood transfusions. Using an Internet‐based vital signs data monitoring system, the heart rate, electrocardiography, respiration rate, and percutaneous oxygen saturation (SpO_2_) were monitored and recorded during the entire home transfusion period.

**Results:**

Ten transfusions in three patients were monitored; two of the patients had an abnormality in a single vital sign (decreased SpO_2_ decrease and increased respiratory rate); these were not transfusion‐related complications. Vital sign anomalies also occur because of errors in using the measurement device and noise associated with body movements. The presence of abnormalities in at least two vital signs among SpO_2_ decrease, tachycardia, and increased respiratory rate that persisted for >5 minutes was defined as a complicated vital sign abnormality (CVSA). There were no severe transfusion‐related complications with CVSA in the present study.

**Conclusion:**

This study indicates the feasibility and sustainability of real‐time remote monitoring of vital signs for the safety of home transfusion. Although CVSA may function as an indicator of severe transfusion‐related complications, these findings need to be confirmed with further studies.

## 1 INTRODUCTION

Home health care is being increasingly administered when specific criteria are met for patients who are frail, chronically ill, or terminally ill.^1^ Home blood transfusion can be a key element in home health care; however, home transfusion programs cannot be fully developed because of limitations related to patient safety and cost.^2^

In blood component transfusion, red blood cells (RBCs), platelets, and plasma that were donated and fractionated are transfused to patients who lack these components. During the blood transfusion, there may be transfusion‐related complications, such as acute hemolysis owing to mismatch of blood types, allergic reactions, and transmission of infectious diseases.^3^ In particular, blood type mismatch and anaphylactic shock are fatal; therefore, careful observation and monitoring of the condition and vital signs after blood transfusion are necessary. In this regard, transfusion therapy is a medical practice that is performed under the supervision of doctors or nurses. It is ideal for a doctor or nurse to be present during the blood transfusion to ensure patient safety. However, in home medical practice, it is difficult for a doctor or nurse to always be present after the start of transfusion until the end, from the viewpoint of human resources.^4^ The guidelines^5^ of the Japan Society of Blood Transfusion and Cell Therapy state that “a patient attendant who will accompany the patient even after the medical staff leaves the patient's home” as an eligibility criterion for performing home blood transfusion.

In the hospital setting, especially in intensive care units, vital signs data are examined using wireless devices. Heart rate, percutaneous oxygen saturation (SpO_2_), and electrocardiograph (ECG) were continuously monitored to detect vital changes; this enables timely responses to changes in vital parameters in a hospital setting. Recently, information and communication technology and remote real‐time monitoring have been developed, resulting in improved home care treatment.^6^ Furthermore, with respect to blood transfusion, real‐time remote monitoring was reportedly performed during blood transfusion.^7^ We extended this method to home care because real‐time monitoring is known to improve the safety of home blood transfusion.

We developed a remote real‐time vital signs data monitoring system for patients with heart diseases.^8^ Using this system, we prospectively remotely monitored patients who were undergoing real‐time blood transfusion at home and investigated whether it is possible to detect abnormal vital signs in addition to transfusion‐related complications from the continuous monitoring data.

## 2 MATERIAL AND METHODS

### 2.1 Patients

Of the patients who underwent home blood transfusions at the Akasaka Clinic (Kobe, Hyogo, Japan) between November 2018 and March 2019, three patients who agreed to participate and gave informed written consent for participation in this study were enrolled. We observed vital signs and patient status during 10 home transfusions. This study was approved by the clinical research review committee at the Wakayama Medical University.

### 2.2 Eligibility criteria for home transfusion

The eligibility criteria for home blood transfusion were established as per the guidelines given by the Japan Society for Transfusion Medicine and Cell Therapy^5^; the main elements included: (a) Home care patients with chronic anemia caused by malignancy and other wasting diseases. (b) Patients who previously underwent transfusion at major hospitals and had no major adverse events because of blood transfusion. (c) Patients understand that home blood transfusion involves a greater risk of transfusion‐related complications than that in the hospital, and (d) an attendant, including family members, is present to oversee the entire procedure of transfusion.

One limitation of home transfusion is that it is not provided for critically ill patients to save the life. Home blood transfusion is provided for chronically ill patients to improve quality of life and prevent organ dysfunction by lack of blood components.

### 2.3 Blood transfusion

Whole blood donations were collected, leukoreduced via filtration, and then fractionated into RBCs and plasma at the Japanese Red Cross Hyogo Blood Center (Kobe, Hyogo, Japan). Blood products were transferred to the Akasaka Clinic (Kobe, Hyogo, Japan) and preserved at 2°C to 6°C in a transfusion refrigerator. At first, the medical staff member collects the patient's blood sample at home. When the decision regarding blood transfusion is taken, the cross‐tested blood unit is transferred in a refrigerated bag by the medical personnel to the patient's home and checked carefully by more than two medical staff members before blood transfusion at the patient's home. Before starting blood transfusion, body temperature, blood pressure, pulse rate, and SpO_2_ were measured and recorded. The doctor then starts blood transfusion by puncturing the peripheral vein or central venous access devices that have been inserted in advance.

### 2.4 Transfusion‐related complications

Transfusion‐related adverse events were categorized into hemolytic reactions, non‐hemolytic reactions, and post‐transfusion infectious diseases.^9^ Hemolytic reactions included major ABO‐incompatible transfusion. The non‐hemolytic reactions included severe allergic reaction, transfusion‐related acute lung injury (TRALI), transfusion‐associated circulatory overload (TACO), and transfusion‐associated graft‐versus‐host disease (TA–GVHD). These severe transfusion reactions were defined as per the International Society of Blood Transfusion.^9^ A major ABO‐incompatible transfusion initially causes fever, tachycardia, tachypnea, reduced blood pressure, renal dysfunction, and may finally cause death. Acute allergic reactions are also observed and cause urticaria, skin rash, and fatal anaphylactic shock in severe cases. TRALI causes hypoxia, and TACO commonly causes heart failure with tachycardia, tachypnea, and hypoxia. Checking and monitoring vital signs, such as heart rate, respiration rate, blood pressure, and O_2_ saturation, are fundamental for detecting the transfusion‐related complications described above.

### 2.5 Vital signs monitoring system

**FIGURE 1:**
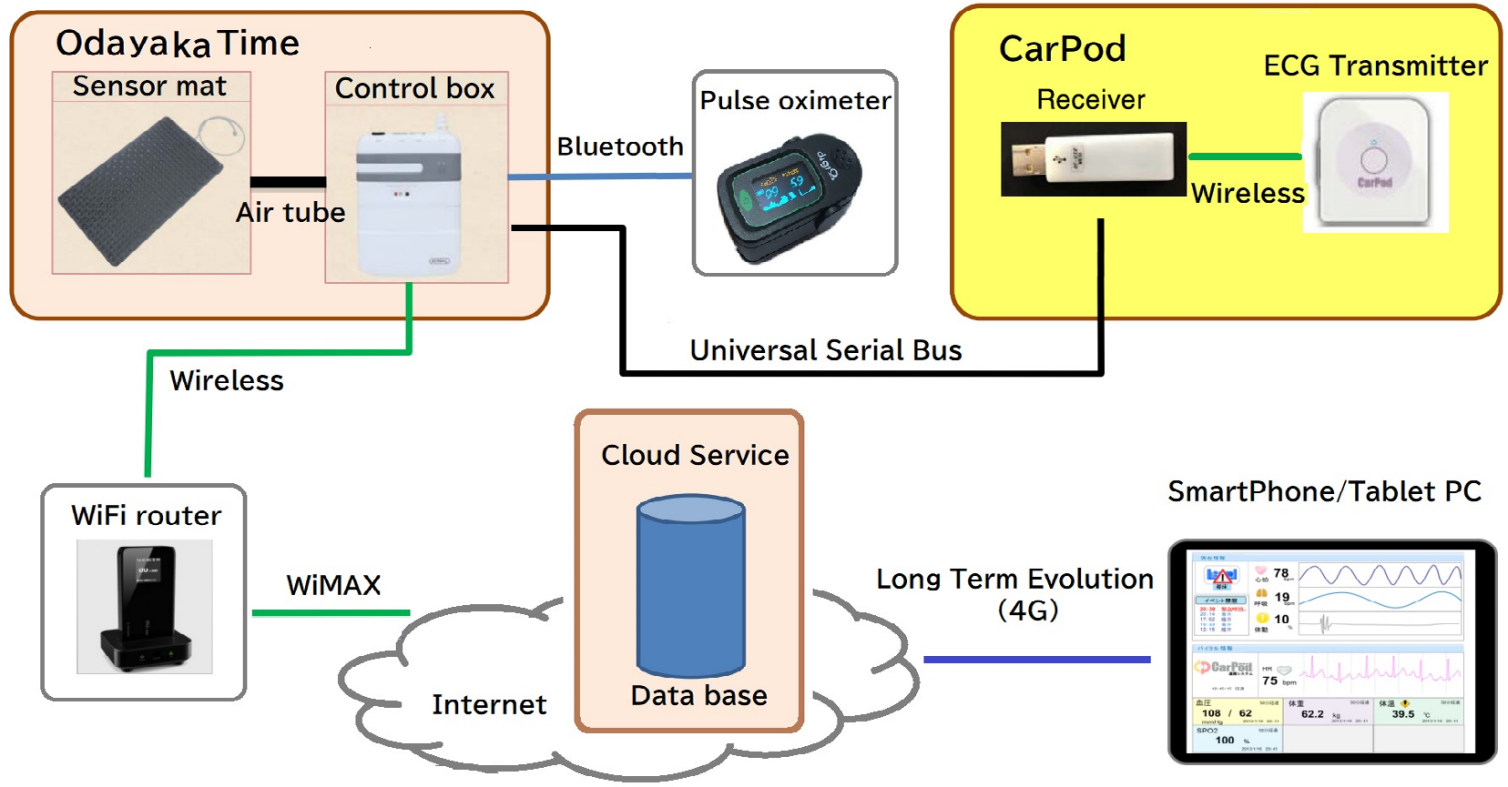
Real‐time remote monitoring system. The system consists of a web‐based remote monitoring subsystem of heart rate and respiration rate, named “Odayaka Time,” a network‐compatible multi‐function portable electrocardiograph “CarPod” and an SpO_2_ monitor with Bluetooth. Measurement data can be monitored in real time on a smartphone/tablet PC from a remote location via the Internet

The remote real‐time vital monitor system includes a web‐based remote monitoring system “Odayaka Time,” a network‐compatible multi‐function portable electrocardiograph “CarPod,” and an SpO_2_ monitor with Bluetooth, as shown in Figure 1. This system was originally developed by Sakano and Kasahara^8^ for observation of patients with heart disease at home. The heart rate can be obtained separately from “Odayaka Time” and “CarPod.” Measurement data can be monitored in real time on a smartphone from a remote location via the Internet.

The system is installed by attaching an electrocardiogram electrode to the chest, wearing an SpO_2_ monitor on the finger, and laying a sensor mat to measure the heart rate, respiratory rate, and body movements on the bed. Vital signs data are uploaded to the Internet in real time via a Wi‐Fi router using portable WiMAX (wireless mobile router). It is not necessary to have a Wi‐Fi connection at home because the physician will bring a portable WiMAX owned by the clinic. The equipment can be easily installed by a physician in approximately 5 minutes, who will make the necessary adjustments at the time of installation, including resolution of Bluetooth connection errors and measurement errors. A doctor can access the Internet using a smartphone and check the patients' vital signs in real time. The equipment attached to the patient include a wireless electrocardiograph, an SpO_2_ monitor, and a sensor mat placed on the bed. Therefore, there are no cords that are physically connected to the patient's body, and they can safely move in the room.

### 2.6 Monitoring procedure

The measurement procedure is as follows: First, the patient is equipped with a remote real‐time vital monitor system, and blood transfusion is started after confirming that the vital signs data have been successfully uploaded to the Internet. The doctor confirms the vital signs remotely and records them on the server. The patient or his/her attendant records the complication details and time and promptly contacts the doctor by phone if transfusion‐related complications develop. The patients' attendants are trained to urgently call a physician and stop the transfusion if they note symptoms that may indicate a respiratory distress or an allergic reaction. In addition, even after leaving the patient's home, doctors continue to provide medical care in the neighborhood so that they can return within 15 minutes if a patient has serious blood transfusion‐related complications. When the attendant calls the doctor, they assess the patients' vital signs on the smartphone and take appropriate action as per the patient's condition. If the patient's condition is serious, the doctor requests for an ambulance, and if it is judged that the condition can be treated at home, the doctor visits the patient's home and treats the patient with anti‐allergic drugs or steroids. The drugs are always available in home‐visit vehicles. If the patient's condition requires oxygen administration, home oxygen therapy can be rapidly introduced. After the blood transfusion is completed, the medical staff measures the vital signs at the patient's home and then removes the needle and the system from the patient.

### 2.7 Monitoring items

The following vital signs were recorded continuously: heart rate, respiratory rate, electrocardiogram, SpO_2,_ and body movements, using the remote real‐time monitor. The details and time of onset of transfusion‐related complications were recorded by the patient or his/her attendant. We compared the timing of abnormal vital signs data during blood transfusion with the timing of changes in the patient's status as recorded by the patient or his/her attendant and confirmed their match rate. Abnormal vital signs were defined as (a) SpO_2_ ≤ 92%, (b) heart rate ≥ 100, or (c) respiratory rate ≥ 22 that continued for >5 minutes. Abnormalities in the vital signs in terms of either a heart rate < 50 or a respiratory rate <10 suggests advanced transfusion complications or monitoring failure, and we examined and interpreted the situation as per the patient's condition. Complicated vital sign abnormality (CVSA) was defined as the presence of >2 of the above (a) to (c) vital abnormalities. CVSA reflects adverse events, such as TRALI, TACO, and anaphylactic shock, as reported previously.^9^

## 3 RESULTS

**FIGURE 2:**
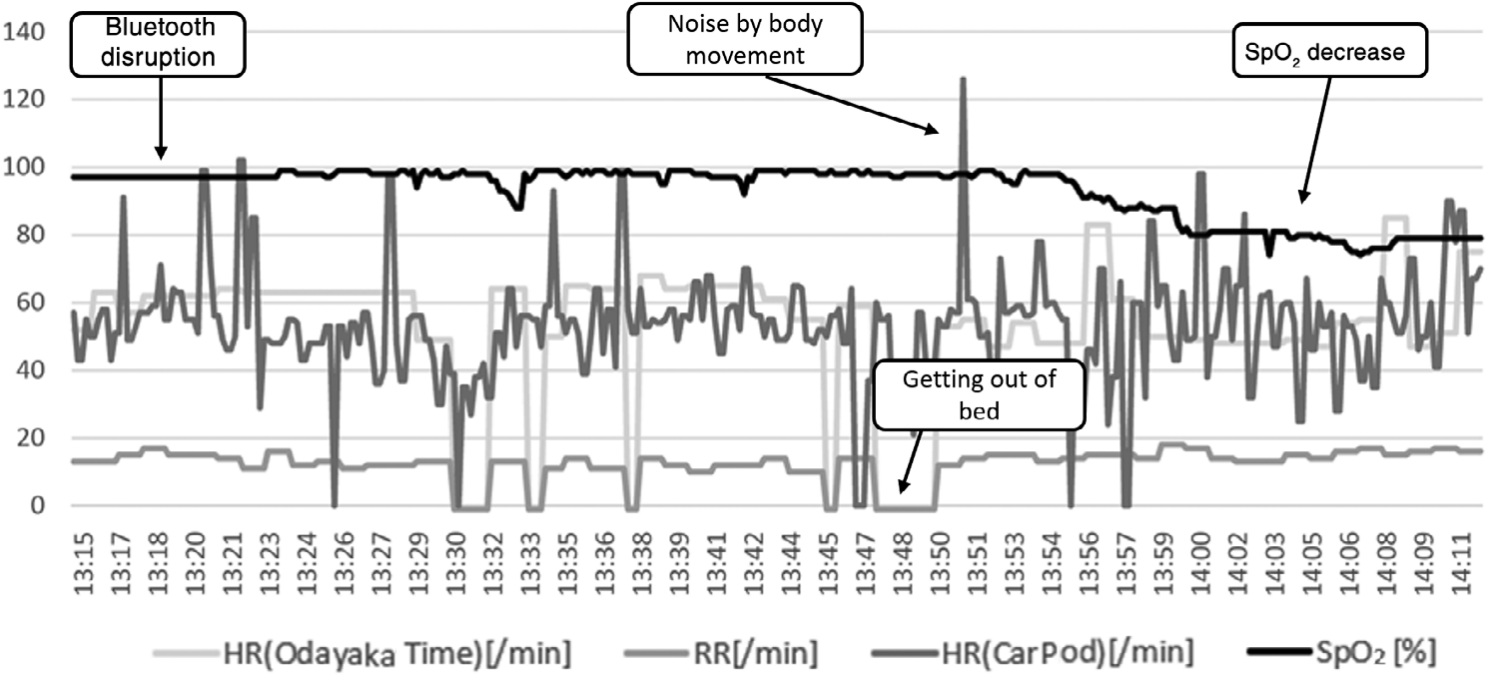
Representative data of the recorded vital signs

We used the Internet‐based vital signs data monitoring system, as shown in Figure 1. A total of 10 home transfusions and remote real‐time monitoring were performed on three patients who provided consent for study participation. These three patients were already undergoing home transfusion therapy; the patients were given an explanation about the study, after which they agreed to participate. The background data of the patients are shown in Table 1. All the patients had hematological malignancies, and it was difficult to perform transfusion at the hospital because of their general condition regarding old age or comorbidities. Representative raw data (monitoring No. 6) are shown in Figure 2. As shown in Table 2, all 10 transfusions included 2 units of RBCs, and the vital signs of abnormality (SpO_2_ ≤ 92%, heart rate ≥ 100, or respiratory rate ≥ 22 that persisted for >5 minutes) were noted in two transfusions (monitoring No. 2 and 6). However, the increase in respiratory rate in No. 2 was considered to be caused by body movements because the patient had chronic lung disease and tachypnea. In monitoring No. 6, SpO_2_ became <80%; however, no change was observed in the patient's condition. We attributed it to measurement trouble or peripheral coldness. As shown in Figure 2, heart rate fluctuation noise because of body movement and data disruption owing to the action of getting out of the bed were observed. Further, as shown in Table 2, there were other measurement errors and equipment trouble. Bluetooth disruption (disruption of the wireless connection between the SpO_2_ monitor and control box), sensor mat malfunction, and disruption of CarPod are involved in the disruption of continuous vital data. Thus, abnormality in a single vital sign or loss did not always reflect abnormalities in the patient's body abnormalities. Therefore, two or more vital sign abnormalities among SpO_2_ decrease, tachycardia, and increased respiratory rate were defined as CVSA. As shown in Table 2, there were no transfusion‐related complications with CVSA.

**TABLE 1:** Patient characteristics

Patient No.	Age	Sex	Disease	Blood product	Units	No. of tests
1	87	F	MDS	RBC	2	8
2	82	F	MM	RBC	2	1
3	64	M	ML	RBC	2	1

*Note*: Two units of RBCs (red blood cells) were derived from 400‐ml whole blood donations.

Abbreviations: MDS, myelodysplastic syndrome; ML, malignant lymphoma; MM, multiple myeloma.

**TABLE 2:** Summary of the results

Monitoring No.	Patient No.	Adverse events	SpO_2_ ≤ 92%	HR ≥ 100	RR ≥ 22	CVSA	Equipment trouble, measurement errors
1	1	None	0	0	0	0	SM
2	2	None	0	0	4^a^	0	SM
3	1	None	0	0	0	0	TBD
4	1	None	0	0	0	0	TBD
5	3	None	0	0	0	0	TBD, DC
6	1	None	2^a^	0	0	0	TBD, DC
7	1	None	0	0	0	0	NE
8	1	None	0	0	0	0	NE
9	1	None	0	0	0	0	NE
10	1	None	0	0	0	0	TBD

Abbreviations: CVSA, complicated vital sign abnormality; DC, disruption of CarPod; HR, heart rate; NE, no measurement error; RR, respiratory rate; SM, sensor mat malfunction; TBD, temporary Bluetooth disruption.

^a^ Frequency of abnormal vital sign during a transfusion.

## 4 DISCUSSION

Recently, remote monitoring of home medical care has advanced owing to the use of implantable cardiac devices, home oxygen therapy, home continuous positive airway pressure therapy, peritoneal dialysis, and other novel medical care devices and methods.^2^ Home blood transfusion is also performed; however, adverse events are the greatest concern in this setting. We set up remote monitoring of vital signs for home blood transfusion to improve the safety and prevent complications related to home blood transfusions. Patients who are eligible to wear a vital monitor are those who have difficulty in accurately communicating their symptoms to their attendants, have a history of minor blood transfusion‐related complications, have heart failure or respiratory failure, and have no attendant.

Adverse events are a major concern when performing blood transfusion. According to the Japanese Society for Transfusion and Cell Therapy Hemovigilance subcommittee, the incidence of pulmonary events (TRALI) in 2016 was 0.1%, that of circulatory overload (TACO) was 0.1%, and that of severe allergic reaction was 0.9%.^10^ In home blood transfusion, patients without a history of serious adverse effects during the blood transfusion are selected based on the guidelines.^5^ Therefore, the incidence of severe adverse effects is expected to be low. At the Akasaka Clinic (Kobe, Hyogo, Japan), where this study was conducted, approximately 2000 home transfusions have been performed during the previous 6 years; however, TRALI, TACO, and anaphylactic shock have not been observed, and only one patient had an allergic reaction associated with platelet transfusion that caused skin rash and itching (unpublished observation). Although the incidence of severe transfusion‐related complications is expected to be low, they can be fatal if not treated appropriately. Therefore, the limitation of home transfusion is that blood transfusion cannot be performed in patients without an attendant, in those with a history of serious blood transfusion‐related complications, in those with serious underlying illness that could be exacerbated by blood transfusion, and in those for whom irregular antibody tests could not be performed correctly (eg, patients taking daratumumab).

Blood transfusion therapy is a medical practice that is usually performed under the supervision of a healthcare professional. In this regard, some home transfusion protocols require a nurse to supervise the entire transfusion procedure.^2^ In other cases, a doctor is also required to stay throughout the process.^11^ In our study, the medical staff was able to leave the patient's home after confirming the patient's stability after transfusion, and the patient's attendant observed the post‐transfusion condition as per the guidelines in Japan.^5^ The safety of this method has also been supported by a large‐scale home study conducted in Italy.^4^ To ensure patient safety during the absence of medical staff members, remote real‐time monitoring of vital signs was introduced in this study.

Changes in vital signs are a prominent feature of transfusion‐related complications. During blood transfusions at the Wakayama Medical University Hospital, respiratory complications of TRALI, including a rapid decrease in the SpO_2_ (SpO_2_ 94% → 89%), an increase in the heart rate (105 bpm → 137 bpm), and a decrease in the blood pressure (systolic blood pressure 130 mm Hg → 80 mm Hg), were observed (unpublished observation). Kamakura^12^ reported that multiple vital sign abnormalities, such as decreased SpO_2_, increased systolic blood pressure, tachycardia, and increased respiratory rate, occurred simultaneously in five TACO analyses. In a report of anaphylactic shock caused by platelet transfusion, Mushino et al^13^ noted that the blood pressure decreased simultaneously with a decrease in SpO_2_. Thus, multiple changes in vital signs reflect transfusion‐related complications. However, as shown in Figure 2 and Table 2, single vital sign anomalies also occur because of device measurement errors and noise associated with body movements. Therefore, the presence of abnormalities in two or more vital signs among SpO_2_ decrease, tachycardia, and increased respiratory rate was defined as CVSA. There were no transfusion‐related complications with CVSA in this study (Table 2). Although CVSA cannot be determined as an indicator of the development of transfusion‐related complications, it is suggested that it helps distinguish between measurement noise and vital changes associated with the patient's body abnormalities. Through further study, CVSA may become an indicator of the development of transfusion‐related complications. In contrast, it is necessary to recognize that even a single vital abnormality may become a potential indicator of mild complications rather than a measurement error.

Home blood transfusions are not performed for patients without an attendant for safety reasons. However, if the monitoring system provides a valid indicator, it may be possible to perform home transfusion safely. Following the indicators, a physician may be able to determine the severity of a patient's condition remotely and decide whether to call an ambulance quickly or treat the patient at his/her home. The frequency of blood transfusion‐related complications is so low that it is not sufficient to discuss the effectiveness of the monitoring system used in this study with a relatively small number of cases. However, it may be meaningful to propose a methodology of using a remote monitoring system for the purpose of safe home transfusion.

Body temperature and blood pressure are also important vital signs; however, currently, there is no simple device that can continuously monitor these parameters in real time. Further, there is no method for measuring these signs other than spot assessment by an on‐site measurer. In all the monitored cases, the body temperature and blood pressure were measured before and after the transfusion; however, no significant abnormality was observed. However, for more accurate monitoring of the patient's condition, the patient's attendant should be trained to measure body temperature and blood pressure at regular intervals and contact a doctor in case of any increase in the value.

## 5 CONCLUSION

In the present study, we developed a monitoring system for home blood transfusion. We found that this system is feasible and stable for remote monitoring of vital signs. Our preliminary results suggest that CVSA is an indicator of transfusion‐related complications. Further studies are necessary to improve the safety of home transfusion using remote real‐time monitoring, including the use of new technologies.

## CONFLICT OF INTEREST

The authors declare that they have no competing interests.

## AUTHOR CONTRIBUTIONS

Conceptualization: Akinori Nishikawa

Data Curation: Akinori Nishikawa

Formal Analysis: Akinori Nishikawa

Funding Acquisition: Yoshihiro Fujimori

Investigation: Akinori Nishikawa

Methodology: Akinori Nishikawa, Yoshihiro Fujimori, Noriko Sakano, Shingo Kasahara

Supervision: Yoshihiro Fujimori, Hiroshi Akasaka, Takashi Sonoki

Writing—Original Draft Preparation: Akinori Nishikawa, Yoshihiro Fujimori

Writing—Review & Editing: Akinori Nishikawa, Yoshihiro Fujimori, Toshiki Mushino, Shinobu Tamura

All authors have read and approved the final version of the manuscript.

Akinori Nishikawa had full access to all of the data in this study and takes complete responsibility for the integrity of the data and the accuracy of the data analysis.

## TRANSPARENCY STATEMENT

The corresponding author affirms that this manuscript is an honest, accurate, and transparent account of the study being reported; that no important aspects of the study have been omitted; and that any discrepancies from the study as planned (and, if relevant, registered) have been explained.

## Data Availability

The datasets used and analyzed during the current study are available from the corresponding author on reasonable request.
